# Machine learning-based QSPR modeling for predicting the *n*-octanol/air partition coefficient of polybrominated diphenyl ethers

**DOI:** 10.1016/j.isci.2026.115393

**Published:** 2026-03-17

**Authors:** Weimin Wu, Hao Chen, Zhaoqin Liu, Wanjun Yan, Daoming Wu, Jingfang Chen, Jun Liu, Zhilin Xing

**Affiliations:** 1 School of Electronic and Information Engineering, Anshun University, Anshun 561000, China; 2 Chongqing Aerospace Polytechnic, Chongqing 400021, China; 3 School of Chemistry and Chemical Engineering, Chongqing University of Technology, Chongqing 400054, China

**Keywords:** environmental science, machine learning, computational materials science

## Abstract

The *n*-octanol/air partition coefficient (*K*_OA_) governs the *trans*-media transport and exposure risk of polybrominated diphenyl ethers (PBDEs). This study utilizes a super learner ensemble—integrating random forest, support vector regression, and multiple linear regression—to predict *K*_OA_ based on density functional theory (DFT) derived quantum-chemical descriptors. This framework estimates properties for 197 PBDE congeners within an applicability domain of log *K*_OA_ 7.24–11.84. SHAP analysis identifies molecular polarizability and the most negative atomic charge as primary determinants, demonstrating that bromination modulates *K*_OA_ via dispersion and electrostatic effects. The ensemble achieves a predictive accuracy of *R*^2^ = 0.981 and reduces extrapolation error by 32%–48% compared to individual algorithms. These findings provide high-precision estimates and mechanistic insights into the environmental fate of PBDEs, supporting hierarchical strategies for monitoring and managing high-mobility congeners effectively.

## Highlights

• RF-SVR ensemble predicts *K*_OA_ for 197 PBDEs (*R*^2^ = 0.981) within log *K*_OA_ 7.24–11.84
• DFT/SHAP show bromination modulates *K*_OA_ via dispersion and electrostatic effects
• The ensemble reduces extrapolation error by 32–48% relative to individual models
• Hierarchical strategy prioritizes risk management for high-mobility PBDE congeners

## Graphical abstract

**Figure:**
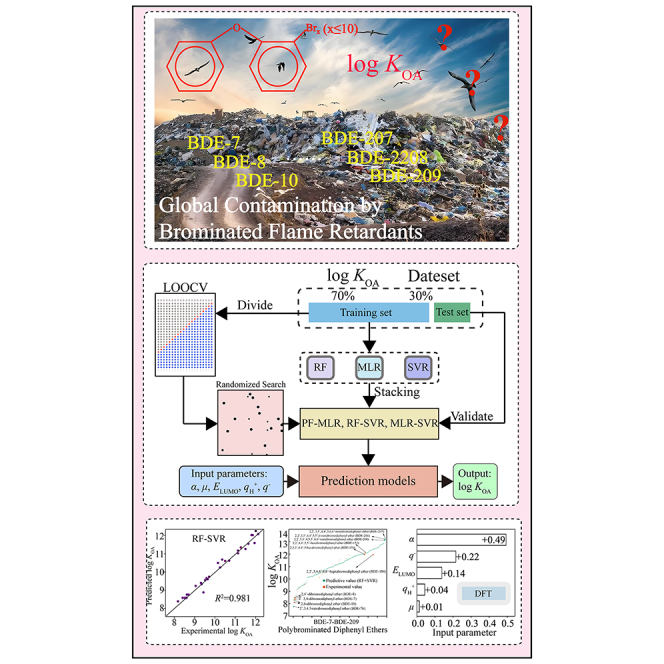

## Introduction

Polybrominated diphenyl ethers (PBDEs), representative brominated flame retardants, were widely utilized in building materials, electronic, furniture, coatings, and plastics owing to their high environmental persistence, long-range transport potential, lipophilicity, and bioaccumulative properties.^1^^,^^2^^,^^3^ However, studies have demonstrated that PBDE metabolites, including hydroxylated (HO-PBDEs) and methoxylated (MeO-PBDEs) derivatives, pose significant threats to ecosystems and human health. These threats manifest through thyroid hormone homeostasis disruption, synaptic transmission inhibition,^4^^,^^5^ and cytochrome P450 enzyme activity interference.^6^^,^^7^ Crucially, a severe scarcity of experimental data for the octanol-air partition coefficient (*K*_OA_), a core parameter governing the environmental fate of PBDEs, was identified. Environmental *K*_OA_ values are available for only approximately 17% of the 209 possible congeners, which significantly hindered the accurate modeling of their *trans*-media transport and risk assessment.

The *K*_OA_ is recognized as a key parameter to characterizing the gas-solid partitioning behavior of semi-volatile organic compounds (SVOCs). It directly governs the dynamic equilibrium of pollutants during processes such as atmospheric particulate adsorption,^8^ plant uptake and enrichment,^9^ and retention by soil organic matter.^10^ While traditional quantitative structure-property relationship (QSPR) models, such as multiple linear regression (MLR), have been employed to predict *K*_OA_ from molecular descriptors, their sensitivity to the complex halogenated structures of PBDEs was found to be inadequate, and capturing nonlinear structure-property relationships proved challenging. In recent years, the application of machine learning (ML) techniques in QSAR/QSPR modeling has expanded rapidly.^11^ ML approaches have significantly enhanced QSPR prediction accuracy by integrating diverse molecular representations, including molecular fingerprints, quantum chemical parameters, and topological descriptors. Nevertheless, single ML models (e.g., random forests (RF) or support vector machines (SVMs)) were observed to exhibit limitations in generalizability and robustness. Notably, extrapolation prediction errors for highly brominated PBDE congeners were reported to reach as high as 1.5–2.0 log units.^12^

To overcome these limitations, ensemble learning (EL) has demonstrated considerable potential. EL leverages the stacking of heterogeneous base models coupled with meta-learner optimization to achieve breakthrough performance.^12^^,^^13^ For instance, Sun et al.^14^ utilized EL to substantially improve the prediction accuracy of QSPR models for the refractive index and viscosity of ionic liquids. Thanh et al.^15^ successfully predicted the corrosion inhibition efficiency of organic compounds using a gradient boosted decision tree (GBDT) combined with the permutation feature importance (PFI) analysis. Similarly, Hu et al.^16^ employed EL to construct a predictive model for Hildebrand solubility parameters. Despite enhanced predictive performance offered by ensemble models, critical aspects require further investigation: the performance variation among different model combinations, and the impact of feature selection strategies on model interpretability and generalization capability. In particular, while tree-based ensembles offer advantages in feature selection, limitations in generalizability and interpretability persisted. Recent advancements have introduced the super learner (a stacking ensemble that learns optimal combinations of base learners from out-of-fold predictions) framework and Stacking generalization techniques,^17^ which further optimize the predictive performance and robustness by strategically fusing diverse model types, such as RF, support vector regression (SVR), and MLR.^18^ However, a predominant focus on performance metrics in existing studies has often neglected the analysis of the environmental behavioral mechanisms driven by key molecular features. This oversight resulted in models functioning as “black boxes,” incapable of providing mechanistic insights into pollutant migration processes.

Against this backdrop, the super learner ensemble architecture was introduced, integrating RF, SVR, and MLR to construct a high-precision *K*_OA_ prediction model for PBDEs. Experimental log *K*_OA_ data were systematically compiled from the literature and integrated with a comprehensive set of multidimensional molecular descriptors (including electronic topological state indices, van der Waals surface areas, and bromine substitution patterns). This integration aimed to reveal the synergistic regulatory mechanism by which bromine substitution sites and molecular polarization effects govern *K*_OA_. Furthermore, the contribution weights of critical molecular features were rigorously quantified using shapley additive explanations (SHAP) and partial dependence plots(PDP) analyses. This approach was designed to transcend the “prediction-focused but explanation-deficient” bottleneck inherent in traditional QSPR modeling. Consequently, this work not only provides a high-accuracy predictive tool for simulating PBDE environmental behavior but also elucidates the physicochemical driving mechanisms of their *trans*-media transport at the molecular interaction level. These insights are expected to lay a theoretical foundation for developing targeted blocking and control technologies for these emerging contaminants.

## Results and discussion

### Model performance evaluation

The QSPR model developed using the super learner (SL) ensemble framework demonstrated significant advantages in predicting the log *K*_OA_ of PBDEs (Table 1). Model performance was evaluated against established criteria (*R*^2^ > 0.7 and Q^2^ > 0.6).^19^ All models exhibited good fit and robustness on the training set, with *R*^2^ values ranging from 0.946 to 0.992, RMSE from 0.227 to 0.429, and MAE from 0.162 to 0.329.

**Table 1:** Performance metrics of individual and ensemble models for log *K*_OA_ prediction

Model	Set	*R*2	RMSE	MAE
RF	Training	0.987	0.429	0.329
	Testing	0.876	0.642	0.492
	Overall	0.943	0.543	0.412
SVR	Training	0.970	0.354	0.276
	Testing	0.882	0.466	0.363
	Overall	0.948	0.410	0.321
MLR	Training	0.971	0.396	0.259
	Testing	0.884	0.521	0.347
	Overall	0.942	0.462	0.301
MLR-SVR	Training	0.976	0.369	0.305
	Testing	0.895	0.463	0.380
	Overall	0.952	0.421	0.342
MLR-RF	Training	0.946	0.227	0.162
	Testing	0.945	0.227	0.162
	Overall	0.946	0.227	0.162
RF-SVR	Training	0.992	0.230	0.208
	Testing	0.951	0.250	0.230
	Overall	0.981	0.240	0.219

Critically, the ensemble models consistently outperformed their constituent single models. The RF-SVR ensemble achieved the highest overall *R*^2^ (0.981), significantly surpassing the best single model (SVR: *R*^2^ = 0.948; MLR: *R*^2^ = 0.942). This underscores the superior capability of the ensemble approach, particularly the RF-SVR combination, in capturing complex nonlinear relationships inherent in the data and enhancing prediction accuracy. Performance remained robust on the independent test set, with *R*^2^ values for all models exceeding 0.7 (ranging from 0.876 to 0.951). Crucially, the MAE values for the test set (0.162–0.329) were consistently within 10% of the corresponding training set MAE ranges, providing strong evidence for the models’ external predictive power and stability.

Ensemble models, especially RF-SVR and MLR-SVR, exhibited notably stronger generalization capabilities on the test set compared to single-algorithm models (RF, SVR, and MLR). These results confirm that the SL framework effectively leveraged the complementary strengths of diverse base learners (MLR, RF, and SVR), leading to substantial improvements in prediction accuracy and model stability. This innovative integration strategy offers a robust solution for QSPR modeling challenges.

### Comparative analysis and advantages of ensemble modeling

**Figure 1:**
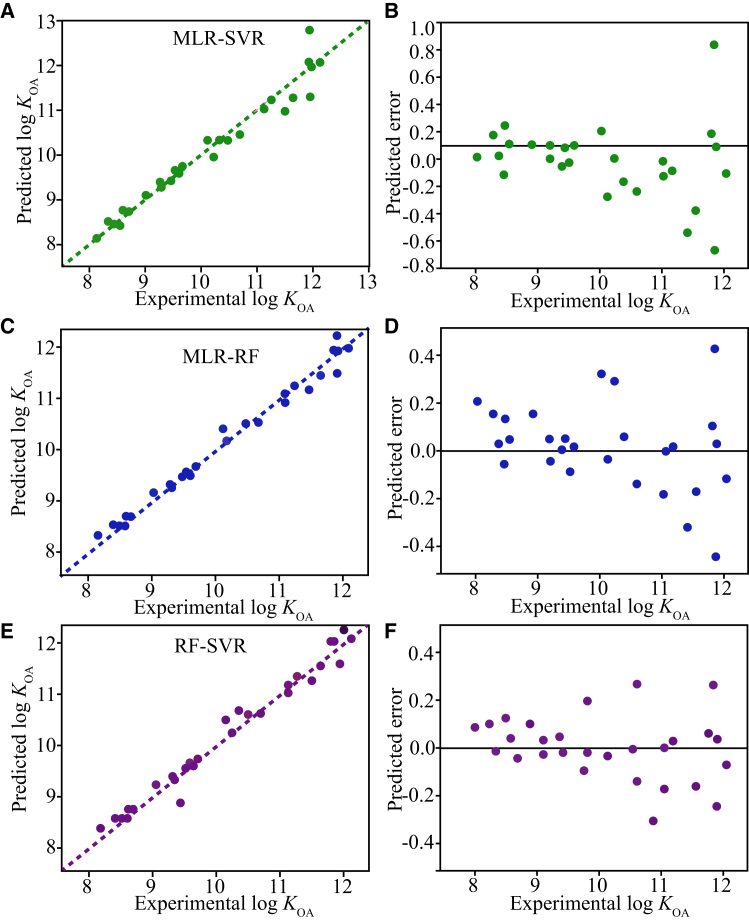
Predicted versus experimental log *K*_OA_ values for all ensemble models (A) Predicted versus experimental log *K*_OA_ values for the MLR-SVR model. (B) Predicted errors versus experimental log *K*_OA_ values for the MLR-SVR model. (C) Predicted versus experimental log *K*_OA_ values for the MLR-RF model. (D) Predicted errors versus experimental log *K*_OA_ values for the MLR-RF model. (E) Predicted versus experimental log *K*_OA_ values for the RF-SVR model. (F) Predicted errors versus experimental log *K*_OA_ values for the RF-SVR model.

A comparative analysis of predicted versus experimental log *K*_OA_ values for all models (Figure 1) further confirmed the superior performance of the ensemble approach. While all models showed some decrease in accuracy on the test set compared to the training set, the ensemble models maintained significantly higher overall prediction accuracy and stability than single models. The RF-SVR ensemble consistently demonstrated the most outstanding performance, achieving the highest test set R^2^ (0.951) and the lowest overall errors (RMSE = 0.240, MAE = 0.219).

The significantly lower RMSE and MAE values observed for the RF-SVR ensemble, compared to both other ensembles and single models (Table 1), validate its superior capability in minimizing prediction error. This highlights a key advantage of the EL strategy: effectively reducing prediction variance and bias, thereby enhancing generalization ability on unseen data. The exceptional performance of RF-SVR across multiple metrics solidifies its position as the optimal model for log *K*_OA_ prediction in this study.

In summary, the comparative analysis unequivocally demonstrates that the SL ensemble framework, particularly the RF-SVR combination, significantly enhances prediction accuracy, robustness, and stability beyond the capabilities of traditional single-algorithm QSPR models.

### PBDEs migration prediction and environmental risk management

The high-precision QSPR ensemble model developed in this study successfully predicted log *K*_OA_ values for PBDEs, providing crucial data for assessing their environmental behavior. The predicted log *K*_OA_ values for the 197 PBDE congeners ranged from 7.24 to 11.84 (mean = 11.21), confirming the strong tendency of these compounds to partition into organic phases (e.g., soil organic matter and lipids) from air. This high affinity indicates significant potential for environmental persistence, long-range transport, and bioaccumulation,^1^^,^^2^^,^^3^^,^^8^^,^^9^^,^^10^ posing substantial risks to ecosystems and human health.

The RF-SVR ensemble model achieved exceptional predictive accuracy (test set *R*^2^ = 0.951, RMSE = 0.230), offering a reliable tool for quantifying PBDE migration characteristics. Chemical industries and environmental regulators can leverage these predictions to implement more effective risk management strategies. For instance, PBDE congeners predicted to have very high log *K*_OA_ values (e.g., >10) could be prioritized for stringent containment measures (e.g., enhanced adsorption technologies or advanced oxidation processes during waste treatment) to minimize environmental release. During the research and development phase of new materials, the model can be used to screen potential PBDE alternatives or assess the environmental fate profile of candidate compounds, promoting the selection of inherently safer chemicals.

The model’s strong performance (training *R*^2^ = 0.992, test MAE = 0.230) and demonstrated generalization ability provide a scientifically robust basis for predicting PBDE environmental transport behavior and informing environmental risk assessment.

### Application domain analysis

**Figure 2:**
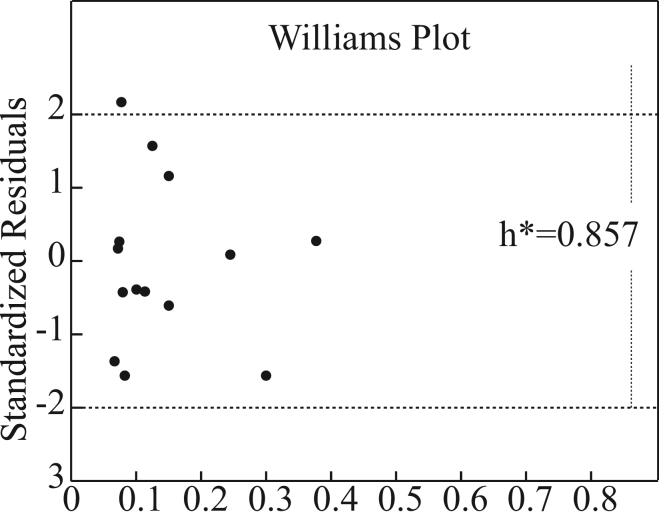
Williams plot assessing the applicability domain of the optimal RF-SVR ensemble model The horizontal dashed lines represent the outlier detection thresholds (standardized residuals = ±2). The vertical dashed line indicates the warning leverage value (h = 0.857). Points represents the chemicals in the dataset.

The applicability domain (AD) of the optimal RF-SVR model was rigorously assessed using Williams plots^20^ (Figure 2). All standardized residuals fell within the acceptable range of ±2, indicating statistically sound predictive accuracy and robustness across the dataset. This confirms that the model not only fits the training data well but also generalizes effectively to the majority of prediction points within the chemical space defined by the training set.

While Figure 2 identifies several high-leverage points (indicating compounds structurally somewhat distinct from the core training set), none exhibited standardized residuals exceeding ±2 or leverage values surpassing the critical threshold ($h^{*}$ = 0.857). These points represent influential but not aberrant data; their inclusion does not adversely affect the model’s overall stability or predictive reliability. Therefore, the Williams plot analysis validates the robustness and high reliability of the RF-SVR ensemble model, confirming its suitability for application within the defined chemical domain (log *K*_OA_ range: 7.24–11.84).

The Williams plot showed that the majority of congeners lay within the warning-leverage threshold and had small standardized residuals, supporting the model’s reliability for most training-like structures. A small set of congeners with extreme bromine substitution exceeded *h*^∗^ or lay outside the descriptor convex hull; these were identified as extrapolations and discussed separately. For these extrapolated congeners, predicted *K*_OA_ values were presented with enlarged uncertainty intervals and interpreted cautiously, and experimental validation was recommended.

### Mechanistic interpretation via SHAP analysis

**Figure 3:**
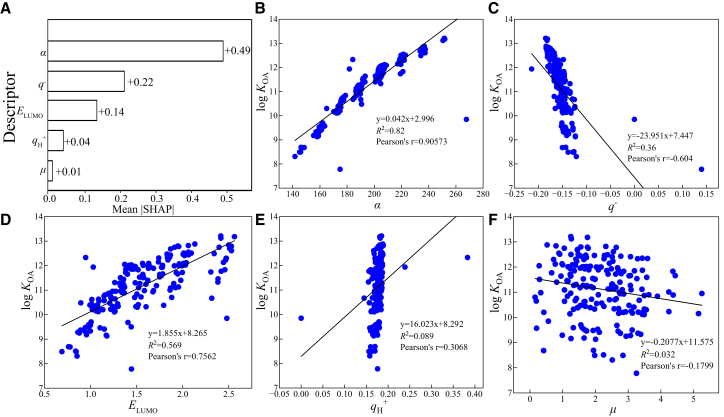
Mean absolute SHAP values for molecular descriptors in the RF-SVR ensemble model, indicating their relative importance for log *K*_OA_ prediction (α, average molecular polarizability, *q*^−^, most negative net atomic charge, *E*_LUMO_, energy of the lowest unoccupied molecular orbital, *q*_H_^+^, most positive net charge on hydrogen atoms; μ: dipole moment) (A) Mean absolute SHAP values of molecular descriptors in the RF-SVR model. (B) Correlation between log *K*_OA_ and α (average molecular polarizability). (C) Correlation between log *K*_OA_ and *q*^−^ (most negative net atomic charge). (D) Correlation between log *K*_OA_ and *E*_LUMO_ (energy of the lowest unoccupied molecular orbital). (E) Correlation between log *K*_OA_ and *q*_H_^+^ (most positive net charge on hydrogen atoms). (F) Correlation between log *K*_OA_ and μ (dipole moment).

To elucidate the decision-making process of the optimal RF-SVR ensemble model and uncover the physicochemical drivers of log *K*_OA_, SHAP analysis^21^ was employed. Figure 3 presents the mean absolute SHAP values, quantifying the relative importance of the five input molecular descriptors. Mean absolute SHAP values were used to provide a robust global ranking of descriptor importance; we did not undertake a full signed-SHAP analysis for global Figures because signed values can be sample-sensitive, and our focus here was on stable global rankings. Signed SHAP/dependence plots will be considered in future work to examine feature directionality in detail.

Average molecular polarizability (*α*) emerged as the dominant feature (mean |SHAP| = 0.49) (Figure 3B). Polarizability governs a molecule’s ability to develop instantaneous dipoles in response to its environment, influencing the strength of London dispersion forces (a major component of van der Waals interactions). For PBDEs, larger molecular volumes and electron clouds (reflected in higher *α*) enhance these attractive interactions with the *n*-octanol phase, thereby increasing log *K*_OA_. This finding is consistent with experimental observations that congeners with higher degrees of bromination (and thus greater polarizability) generally exhibit higher *K*_OA_ and stronger partitioning into organic phases.

The most negative net atomic charge (*q*^−^) was the second most influential descriptor (mean |SHAP| = 0.22). This feature identifies the most electronegative atom(s) in the molecule, which are potential sites for specific electrostatic interactions (e.g., dipole-dipole and hydrogen bonding if H-bond acceptors are present) or polarization effects. For PBDEs, bromine atoms often carry significant partial negative charges, influencing their interaction with polar groups in *n*-octanol or other environmental matrices. Thus, *q*^−^ captured the tendency of bromine-substituted sites to modulate adsorption and local polarization, aligning with reported patterns where electronic distribution around halogen substituents affected PBDE adsorption and phase affinity.

To illustrate these effects with concrete examples, we compared congeners with contrasting substitution patterns. For example, BDE-1 (monobrominated) displayed low *α* and a modest *q*^−^ at halogen sites and was predicted (and experimentally observed) to have one of the lowest log *K*_OA_ values in our dataset, consistent with weak dispersion-driven affinity for *n*-octanol. In contrast, heavily brominated congeners such as BDE-183 (and other hexa-to-decabrominated congeners) exhibited much larger *α* and more pronounced negative partial charges on bromine sites; the model therefore assigned substantially higher predicted log *K*_OA_ to these congeners, reflecting stronger van der Waals attraction and enhanced local polarization. An intermediate case, BDE-47 (tetra-brominated), showed moderate *α* and *q*^−^ values and correspondingly intermediate predicted *K*_OA_, illustrating a graded, monotonic influence of bromination level on the polarizability-driven partitioning behavior.

The contributions of the other descriptors were comparatively lower: energy of the LUMO (*E*_LUMO_) (mean |SHAP| = 0.14), related to electron affinity and potential for charge-transfer interactions; most positive net charge on hydrogen atoms (q_H_^+^) (mean |SHAP| = 0.04), potentially relevant if H-bond donating capability exists; and dipole moment (*μ*) (mean |SHAP| = 0.01), reflecting overall molecular polarity. While less globally important, these features may contribute to predictions for specific subsets of congeners.

In summary, SHAP analysis revealed that log *K*_OA_ prediction for PBDEs is primarily governed by molecular polarizability (*α*), dictating non-polar van der Waals interactions, and the most negative net charge (*q*^−^), influencing polar/electrostatic interactions. The specific-congener examples above corroborated how these descriptors translated into the model’s rankings and predictions, thereby linking quantum chemical descriptors to experimentally observed partitioning behavior and demonstrating that the model captured chemically meaningful drivers rather than spurious correlations.

### Comparative analysis with existing models

The performance of the optimal RF-SVR ensemble model was benchmarked against representative QSPR models for log *K*_OA_ prediction reported in the literature (Table 2). Zhao et al.^22^ constructed a QSPR model based on the data of *K*_OA_ of 19 hydroxylated polybrominated diphenyl ethers (OH-PBDEs) and 10 methoxylated polybrominated diphenyl ethers (MeO-PBDEs) by partial least squares (PLS) analysis coupled with molecular description, with a coefficient-of-determination (CoD) value of 0.961. Long et al.^23^ constructed a model based on the log *K*_OA_ data of 22 PBDEs by MLR and artificial neural network (ANN) combined with molecular distance edge vector (MDEV) indices, respectively, with a prediction accuracy of 0.973. Liu et al.^24^ extracted spatial, electrostatic, hydrophobic, and hydrogen-bonding by performing molecular similarity index analysis (CoMSIA) on 19 PBDEs related descriptors, and constructed a QSPR model using PLS with an accuracy of 0.952.

**Table 2:** Performance comparison of the present RF-SVR ensemble model with representative literature models for PBDE log *K*_OA_ prediction

References	Dataset size	Model	Accuracy	Application domain
Zhao et al.^22^	29 (OH/MeO-PBDEs)	PLS	0.961	No
Long et al.^23^	22 PBDEs	ANN/MLR	0.973	No
Li et al.^24^	19 PBDEs	PLS (CoMSIA)	0.952	No
This Study	30 PBDEs	RF-SVR Ensemble	0.981	Yes

While these studies demonstrated good predictive performance within their respective datasets, the RF-SVR ensemble model developed in this study achieved a significantly higher overall *R*^2^ (0.981) using a dataset of comparable size (30 PBDEs). Crucially, this study advances the field in several key aspects. Enhanced Methodology: The application of the super learner ensemble framework integrating diverse ML algorithms (RF, SVR, and MLR) represents a methodological advancement over single-model approaches (PLS, MLR, and ANN) used in previous studies.

First, the application of the super learner ensemble framework integrating diverse ML algorithms (RF, SVR, and MLR) represents a methodological advancement over the single-model approaches (PLS, MLR, and ANN) typically employed in previous studies. This integrated strategy offers superior predictive capability.

Second, the study provides stronger evidence for model robustness and reliable applicability through rigorous validation using leave-one-out cross-validation (LOOCV) and an independent test set, coupled with explicit AD characterization via Williams plots. This contrasts with studies lacking explicit AD definition or extensive validation, as indicated by “none” in Table 2.

Third, moving beyond purely predictive models, the integration of SHAP interpretability analysis delivers unprecedented insight into the molecular features and physicochemical interactions governing log *K*_OA_. This mechanistic understanding significantly enhances the model’s scientific value.

### Practical significance and future directions

**Figure 4:**
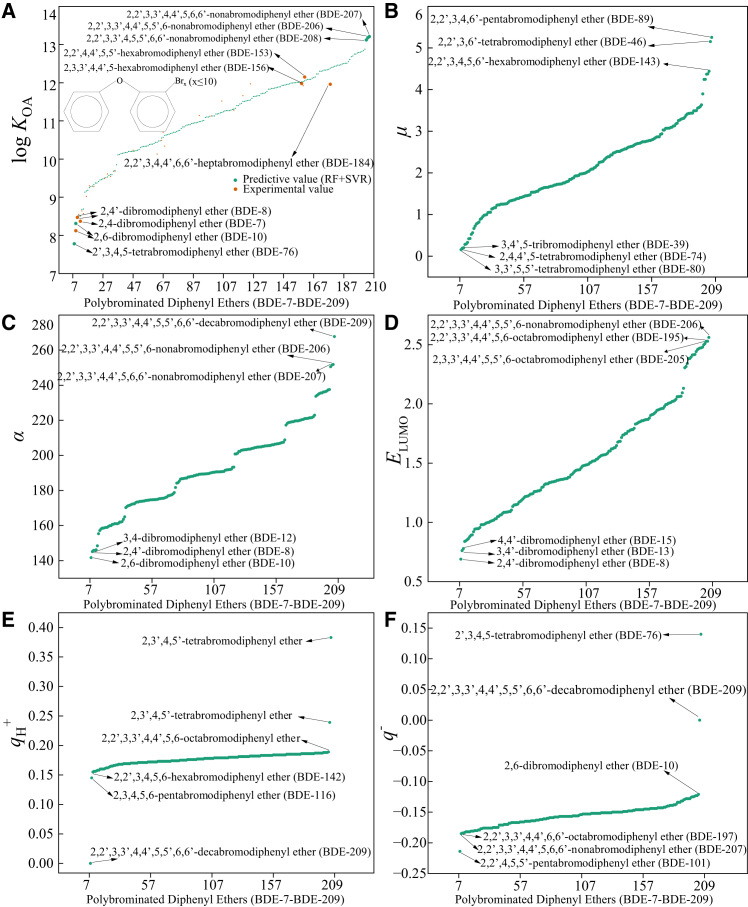
Predicted and structural descriptor variations of polybrominated diphenyl ethers (PBDEs, BDE-7 to BDE-209) in the RF-SVR ensemble model The figure illustrates the trends of predicted and experimental log *K*_OA_ values and their relationships with key molecular descriptors (*μ*, *α*, *E*_LUMO_, *q*_H_^+^, and *q*^−^). (A) Predicted versus experimental log *K*_OA_ values of PBDEs in the RF-SVR model. (B) Variation trend of *μ* (dipole moment) across PBDE congeners. (C) Variation trend of *α* (average molecular polarizability) across PBDE congeners. (D) Variation trend of *E*_LUMO_ (energy of the lowest unoccupied molecular orbital) across PBDE congeners. (E) Variation trend of *q*_H_^+^ (most positive net charge on hydrogen atoms) across PBDE congeners. (F) Variation trend of *q*^−^ (most negative net atomic charge) across PBDE congeners.

Figure 4 (Panels A–F) summarizes the ensemble-model predictions and selected quantum-chemical descriptors across the full set of PBDE congeners examined. Panel A compared predicted and available experimental log *K*_OA_ values and showed that congeners spanned a wide partitioning space (approximately log *K*_OA_ ≈ 7–13), with a pronounced subset of highly brominated congeners exhibiting very large log *K*_OA_ (many >10). Panels B-F plotted the same congener ordering against dipole moment (*μ*), molecular polarizability (*α*), *E*_LUMO_, and site-specific partial charges (*q*_H_^+^ and *q*^−^). These plots demonstrated systematic trends: Molecular polarizability increased markedly with bromination and showed the strongest positive association with log *K*_OA_, whereas the most negative atomic partial charge (*q*^−^) produced a consistent secondary modifying effect. Dipole moment, *E*_LUMO_, and *q*_H_^+^ exerted smaller, congener-specific influences. The patterns visible in Figure 4 were consistent with model interpretability analyses and reinforced that dispersion-dominated interactions (increased van der Waals/polarizability effects with bromine loading) constituted the primary molecular basis for elevated air-organic partitioning in PBDEs, while electronic-structure features modulated that baseline behavior.

From a practical-use perspective, the magnitude and distribution of predicted log *K*_OA_ had direct implications for fate, transport, and exposure pathways and were translated into concrete actions for decision makers and practitioners. Congeners with very high predicted log *K*_OA_ were identified as having a strong propensity to partition into organic phases (soil and sediment organic matter, atmospheric particulate matter, and biological lipids), which implied increased persistence, bioaccumulation potential, and particle-associated long-range atmospheric transport. In response to the reviewer’s request for realistic application pathways, experts recommended that the model outputs were used to (1) implement rapid screening and prioritization—flagging congeners above a pre-defined log *K*_OA_ threshold (for example, >10) for immediate monitoring and further testing; (2) focus scarce laboratory resources by selecting high-risk congeners for targeted experimental *K*_OA_ determination and bioaccumulation assays; (3) inform regulatory grouping and substitution decisions by identifying congeners whose partitioning behavior rendered them most likely to persist and bioaccumulate; (4) guide environmental monitoring design and sampling strategies (e.g., emphasizing particle-associated sampling in regions where high- *K*_OA_ congeners were predicted to dominate); and (5) support remediation and treatment planning by indicating when technologies that address particle-bound or strongly sorbed fractions were required. It was also recommended that model predictions were integrated into screening-level multimedia fate and exposure models to estimate relative risk across compartments and life cycle stages, and that cost-benefit analyses used the prioritized list to allocate monitoring and mitigation funding efficiently.

Finally, it was noted that utility for policy and practice depended on the transparent communication of model uncertainty and applicability limits. Therefore, experts recommended that any policy application be accompanied by (1) explicit applicability-domain checks and uncertainty bounds on individual predictions, (2) a short list of congeners prioritized for near-term experimental validation, and (3) documented decision rules (e.g., how a log *K*_OA_ threshold was selected and how predictions triggered monitoring or regulatory actions). These measures were intended to ensure that the model outputs were used as an evidence-based, cost-effective triage tool—directing laboratory effort, field surveillance, and regulatory attention to the congeners most likely to pose long-term environmental and human-health risks.

### Limitations of the study

While the present study demonstrates the effectiveness of the super learner ensemble framework in predicting the log *K*_OA_ of PBDEs, several limitations should be acknowledged. First, the experimental dataset used for model development comprised only 30 *K*_OA_ values, which is relatively small compared to the 197 PBDE congeners considered. This limitation may affect the model’s generalizability, particularly for extrapolative predictions beyond the chemical space defined by the training set. To mitigate this, we employed physics-based density functional theory (DFT) descriptors to enhance mechanistic interpretability and transferability, implemented an EL strategy to reduce model variance, and conducted rigorous validation, including LOOCV and independent test set evaluation. Additionally, the AD was explicitly characterized using Williams plots to identify and flag predictions that may lie outside the reliable prediction domain. *Full Williams* plots, the warning-leverage threshold calculations (h), and multivariate distance metrics (e.g., Mahalanobis distances) were reported in the supplemental information. Despite these precautions, users are advised to interpret out-of-domain predictions with caution.

Second, alternative validation approaches such as Y-scrambling and external validation were not applied in this study, primarily due to the limited availability of experimental *K*_OA_ data for PBDEs. Nevertheless, model robustness was ensured through 10-fold cross-validation and an independent 70:30 train-test split, both of which demonstrated strong predictive performance and stability. This limitation is fully acknowledged, and future studies will incorporate Y-scrambling and external validation using newly available experimental datasets to further confirm model reliability.

Third, the selection of molecular descriptors, though grounded in quantum chemical calculations, may not fully capture all relevant intermolecular interactions influencing *K*_OA_. While SHAP analysis provided insights into key features such as polarizability and electrostatic properties, other structural or conformational factors not included in the current descriptor set could further refine predictions.

Finally, the model’s performance, though superior to existing approaches, still relies on the quality and representativeness of the limited experimental data. Future expansion of the experimental *K*_OA_ database for PBDEs and related compounds will be essential to enhance model robustness and broaden its applicability.

## Resource availability

### Lead contact

Further information and requests for resources, data, or code should be directed to and will be fulfilled by the lead contact, Zhilin Xing (xingzhilin@cqut.edu.cn).

### Materials availability

No new experimental materials or patented compounds were generated in this study. All computational and model-derived data were obtained from quantum chemical calculations and ML analyses described in the paper and supplemental information.

### Data and code availability

• Data reported in this paper will be shared by the lead contact upon request.
• This paper does not report original code.
• Any additional information required to reanalyze the data reported in this paper are available from the lead contact upon request.

## Acknowledgments

This study was funded by the Anshun University Doctoral Special Fund Project (no. anxybsjj202411), the Youth Scientific and Technological Talent Cultivation Project of “Two Cities and Three Bases” of Anshun Municipal Science and Technology Bureau (no. anshunshikeren-2024-12-17-01), the 10.13039/501100001809National Natural Science Foundation of China (no. 52200145), the Chongqing Technology Innovation and Application Development (Key Project) (no. CSTB2023TIAD-KPX0071), and the Science and Technology Research Program of Chongqing Education Commission of China (no. KJQN202201131). Additionally, we would like to thank SCl-GO Test (www.sci-go.com) and Home for Researchers (https://www.home-for-researchers.com) for their services.

## Author contributions

Investigation, formal analysis, writing-original draft, and funding acquisition: W.W.; project administration and writing-original draft: H.C.; investigation and validation: Z.L.; methodology and resources: W.Y.; data curation and visualization: D.W.; validation and writing – review and editing: J.C.; conceptualization and investigation: J.L.; funding acquisition, supervision, and writing – review and editing: Z.X.

## Declaration of interests

The authors declare no competing interests.

## Declaration of generative AI and AI-assisted technologies in the writing process

During the preparation of this work, the author(s) used ChatGPT in order to improve the readability and language of the manuscript. After using this tool/service, the author(s) reviewed and edited the content as needed and take(s) full responsibility for the content of the published article.

## STAR★Methods

### Key resources table

**Table:** 

REAGENT or RESOURCE	SOURCE	IDENTIFIER
**Deposited data**		

Experimental log *K*_OA_ values	Zhao et al.^25^; Wania et al.^26^	This paper, Table S1
Predicted log *K*_OA_ values (167 congeners)	This paper	This paper, Table S2

**Software and algorithms**		

Gaussian 16	Gaussian, Inc.	https://gaussian.com
Python (v3.8 or higher)	Python Software Foundation	RRID: SCR_008394
Scikit-learn	Pedregosa et al.^27^	RRID: SCR_002577
Pandas	The pandas development team	RRID: SCR_018337
Numpy	Harris et al.^28^	RRID: SCR_008633
Scipy	Virtanen et al.^29^	RRID: SCR_008058
SHAP (shapley Additive explanations)	Lundberg et al.^30^	https://github.com/slundberg/shap
Matplotlib	Hunter^31^	RRID: SCR_008624
Seaborn	Waskom et al.^32^	RRID: SCR_018132
Scripts for RF-SVR Stacking and SHAP	This paper	Available from lead contact upon request

### Method details

#### Data sources and pre-processing

The theoretical scope included 197 PBDEs congeners and isomers with 1–10 bromine substitutions. Model development relied on experimentally determined *K*_OA_ values for 30 PBDEs congeners at 298.15 K (25 °C). Records with incompatible experimental conditions were excluded. Experimental log *K*_OA_ values compiled from literature,^25^^,^^26^ ranged from 7.24 (BDE-1) to 11.84 (BDE-184).

To ensure representativeness, multiple measurements for the same congener were averaged. The mean (*μ*) log *K*_OA_ value was 11.21, with a standard deviation (*σ*) of 1.40. All values fell within *μ* ± 3*σ*; according to the Pauta Criterion, no outliers were identified. The dataset was randomly partitioned into training and validation sets at a 7:3 ratio. Principal Component Analysis (PCA)^33^ was applied to reduce dimensionality. Descriptor data were normalized via *Z* score standardization (mean of 0, standard deviation of 1) to mitigate scaling issues.

#### Molecular descriptor calculation and screening

The selection and generation of molecular descriptors were recognized as critical factors influencing the predictive performance and interpretability of QSPR models.^34^^,^^35^ In this study, descriptors were calculated using Gaussian 16.^36^ Geometric optimization was conducted at the B3LYP/6-31G(d) level using DFT with Grimme dispersion correction D3(BJ) to account for halogenated aromatic interactions. Tightened numerical settings (Opt = Tight, SCF = VeryTight, Int = UltraFine) were used to ensure accurate gradients. All optimized structures were validated by harmonic vibrational frequency analysis (Freq) to confirm the absence of imaginary frequencies.

Based on mechanistic relevance and empirical screening (including correlation and LASSO analysis), five quantum chemical descriptors were selected: (1) average molecular polarizability (*α*); (2) dipole moment (*μ*); (3) energy of the lowest unoccupied molecular orbital (*E*_LUMO_); (4) most positive net charge on the hydrogen atoms (*q*_H_^+^); and (5) most negative net charge (*q*^−^). Values were extracted from Gaussian 16 output files. These descriptors capture polarizability, electronic properties, and local charge sites. Atomic charges were extracted via Natural Population Analysis (NPA/NBO) routines.

#### Ensemble learning architecture and training

An ensemble learning strategy^37^ was adopted using Stacking. The framework consisted of two levels.^38^ Level-0 (Base Learners) included Random Forest (RF),^39^ Support Vector Regression (SVR), and Multiple Linear Regression (MLR). RF was utilized for its robustness against noisy descriptors, while SVR effectively modeled smooth, high-dimensional nonlinearities via kernel functions. MLR provided an interpretable linear baseline. These were trained on the original data. Level-1 (Meta-Learner) used predictions from Level-0 as input features to generate the final prediction (Figure S1).

Level-1 (Meta-Learner) used predictions from Level-0 as input features to generate the final prediction. Pairing tree-based learners with kernel or linear models increases ensemble diversity. Weights for the ensemble were determined by an inverse-error rule based on repeated cross-validated RMSE. The RF-SVR pairing was identified as the optimal ensemble for reporting.

#### Model training and hyperparameter tuning

Hyperparameter optimization utilized a Leave-One-Out Cross-Validation (LOOCV) strategy (Figure S2).

**RF:** Optimized for number of estimators and maximum tree depth via Grid Search.^40^

**MLR:** Regularization strength (*α*) was optimized using Ridge^41^ or Lasso^42^ regression.

**SVR:** Optimized via Random Search^43^ for penalty parameter (*C*), kernel type (RBF), kernel coefficient (*γ*), and error tolerance (*ε*).

### Quantification and statistical analysis

#### Statistical metrics and software

Model performance was evaluated using the Coefficient of Determination (*R*^2^), Root-Mean-Square Error (RMSE), and Mean Absolute Error (MAE).

(Equation 1) $$\begin{array}{c} R^{2}=1-\frac{\sum_{i=1}^{n}{\left(Y_{i}-{\hat{Y}}_{i}\right)}^{2}}{\sum_{i=1}^{n}{\left(Y_{i}-\bar{Y}\right)}^{2}} \end{array}$$

(Equation 2) $$\begin{array}{c} \text{RMSE}=\sqrt{\frac{1}{n}\sum_{i=1}^{n}{\left(Y_{i}-{\hat{Y}}_{i}\right)}^{2}} \end{array}$$

(Equation 3) $$\begin{array}{c} \text{MAE}=\frac{\sum_{i=1}^{n}\left|Y_{i}-{\hat{Y}}_{i}\right|}{n} \end{array}$$

where *Y*_i_ is the experimental observation, ${\hat{Y}}_{i}$ is the model prediction, $\bar{Y}$ is the mean of the experimental values, and *n* = 30 is the number of PBDE congeners with experimental data. All statistical analyses and machine learning modeling were performed using Python (version 3.8). The following software libraries were used: scikit-learn (v1.0.2) for model training and hyperparameter optimization, SHAP (v0.40.0) for interpretability analysis, NumPy and Pandas for data manipulation, and Matplotlib and Seaborn for data visualization.

#### Validation and interpretability

Leave-One-Out Cross-Validation (LOOCV)^44^ was employed to assess generalization. A single sample was held out as the validation set while *n*-1 samples were used for training; this was repeated n times. The applicability domain (AD) was defined using Williams plots (standardized residuals versus leverage values) to identify extrapolative predictions. The leverage warning value *h*∗ was set at 3*p*/*n* (where *p* is the number of descriptors).

Model interpretability was provided by SHAP (SHapley Additive exPlanations). Local attributions were computed for each congener and aggregated globally via mean absolute SHAP. TreeSHAP was used for RF, and KernelSHAP for SVR and MLR. Ensemble-level feature importance was calculated as a weighted average of Level-0 importances using inverse-RMSE weights.

### Additional resources

This study did not involve clinical trials; therefore, no clinical registry information is reported.

## Supplemental information

Document S1. Figures S1, S2, Table S1, and Methods S1

Table S2. Predicted log *K*_OA_ values for 167 PBDE congeners calculated by the optimized RF-SVR ensemble model, related to Figure 4
