# Supplementary material for: Machine learning-based QSPR modeling for predicting the n-octanol/air partition coefficient of polybrominated diphenyl ethers
Source: iScience. 2026 Mar 17;29(4):115393. doi: 10.1016/j.isci.2026.115393 (PMC13068587; doi:10.1016/j.isci.2026.115393)
Supplement: Document S1. Figures S1, S2, Table S1, and Methods S1 [file mmc1.pdf]

iScience, Volume *29*

Supplemental information

Machine learning-based QSPR modeling for predicting the *n*-octanol/air partition coefficient of polybrominated diphenyl ethers

Weimin Wu, Hao Chen, Zhaoqin Liu, Wanjun Yan, Daoming Wu, Jingfang Chen, Jun Liu, and Zhilin Xing

Methods S1. Methods S1. Summary of hyperparameter search spaces and convergence metrics, related to STAR Methods.

Model	Hyperparameter(s)	Search range / options	Final selected value(s)	Search method	CV settings	Notes
Random Forest (RF)	n_estimators; max_depth	100–1000; {None,5,10,20,30,50}	n_estimators = 200; max_depth = 20	Grid Search	repeated 5-fold CV (5 repeats)	Grid used for tree parameters
MLR (Ridge/Lasso tested)	model type; alpha	OLS / Ridge / Lasso; α ∈ [1e-4, 1e2]	Ridge; α = 1	Grid Search	repeated 5-fold CV (5 repeats)	Ridge chosen
Support Vector Regression (SVR)	C; gamma; epsilon; kernel	C ∈ [1e-3,1e3] (log); γ ∈ [1e-4,1e1] (log) or 'scale'/'auto'; ε ∈ [1e-4,1.0]; kernels {RBF, lin, poly}	C = 10; gamma = 'scale'; epsilon = 0.1; kernel = RBF	Randomized Search (100 iterations)	Nested CV (outer 5-fold / inner 5-fold); final repeated 5-fold x5	Random search for SVR hyperparameters

Table S1 Experimental log K_{OA} values (measured at 298.15 K) for 30 PBDE congeners

No.	Abbreviation	Polybrominated Diphenyl Ethers	Molecular Formula	Experimental Value	MLR	SVM	RF	MLR+SVM	MLR+RF	RF+SVM	u	α	Elumo	qH+	q-
1	BDE-7	2,4-dibromodiphenyl ether	$C_{12}H_8Br_2O$	8.37	8.55	8.53	8.50	8.54	8.52	8.51	2.251	145.820	0.8386	0.170	-0.126
2	BDE-8	2,4'-dibromodiphenyl ether	$C_{12}H_8Br_2O$	8.47	8.50	8.48	8.51	8.49	8.50	8.49	2.843	145.134	0.6895	0.156	-0.138
3	BDE-10	2,6-dibromodiphenyl ether	$C_{12}H_8Br_2O$	8.12	8.14	8.12	8.50	8.13	8.32	8.31	2.516	141.701	0.8533	0.158	-0.121
4	BDE-12	3,4-dibromodiphenyl ether	$C_{12}H_8Br_2O$	8.55	8.44	8.42	8.57	8.43	8.50	8.49	1.980	145.817	0.8446	0.162	-0.128
5	BDE-13	3,4'-dibromodiphenyl ether	$C_{12}H_8Br_2O$	8.57	8.82	8.81	8.58	8.81	8.70	8.69	1.941	146.168	0.7616	0.170	-0.143
6	BDE-15	4,4'-dibromodiphenyl ether	$C_{12}H_8Br_2O$	8.64	8.76	8.74	8.62	8.75	8.69	8.68	0.419	148.458	0.7779	0.160	-0.150
7	BDE-17	2,2',4-tribromodiphenyl ether	$C_{12}H_7Br_3O$	9.30	9.31	9.30	9.20	9.30	9.26	9.25	2.939	158.785	0.8783	0.171	-0.137
9	BDE-21	2,3,4-tribromodiphenyl ether	$C_{12}H_7Br_3O$	9.49	9.43	9.42	9.58	9.43	9.50	9.50	3.124	160.589	1.4421	0.166	-0.128
16	BDE-28	2,4,4'-tribromodiphenyl ether	$C_{12}H_7Br_3O$	9.54	9.62	9.62	9.56	9.62	9.59	9.59	1.325	161.993	0.9811	0.172	-0.151
18	BDE-30	2,4,6-tribromodiphenyl ether	$C_{12}H_7Br_3O$	9.02	9.13	9.12	9.21	9.13	9.17	9.16	1.255	158.936	1.1710	0.176	-0.123
20	BDE-32	2,4',6-tribromodiphenyl ether	$C_{12}H_7Br_3O$	9.28	9.39	9.38	9.28	9.38	9.33	9.33	3.385	157.140	0.9980	0.160	-0.148
23	BDE-35	3,3',4-tribromodiphenyl ether	$C_{12}H_7Br_3O$	9.61	9.58	9.57	9.49	9.58	9.53	9.53	2.073	159.532	0.9863	0.179	-0.144
25	BDE-37	3,4,4'-tribromodiphenyl ether	$C_{12}H_7Br_3O$	9.68	9.77	9.77	9.63	9.77	9.70	9.70	1.556	161.996	1.0059	0.179	-0.151
35	BDE-47	2,2',4,4'-tetrabromodiphenyl ether	$C_{12}H_6Br_4O$	10.34	10.34	10.34	10.90	10.34	10.62	10.62	1.654	175.714	1.0146	0.173	-0.160
54	BDE-66	2,3',4,4'-tetrabromodiphenyl ether	$C_{12}H_6Br_4O$	10.49	10.32	10.32	10.79	10.32	10.55	10.55	2.039	175.362	1.0907	0.174	-0.155
57	BDE-69	2,3',4,6-tetrabromodiphenyl ether	$C_{12}H_6Br_4O$	10.23	9.96	9.95	10.45	9.95	10.20	10.20	0.425	172.195	1.2591	0.177	-0.142
63	BDE-75	2,4,4',6-tetrabromodiphenyl ether	$C_{12}H_6Br_4O$	10.13	10.33	10.33	10.55	10.33	10.44	10.44	1.691	174.763	1.2944	0.177	-0.150
65	BDE-77	3,3',4,4'-tetrabromodiphenyl ether	$C_{12}H_6Br_4O$	10.70	10.46	10.47	10.67	10.46	10.57	10.57	2.193	175.366	1.1484	0.179	-0.156
70	BDE-82	2,2',3,3',4-pentabromodiphenyl ether	$C_{12}H_5Br_5O$	11.14	11.01	11.02	10.94	11.01	10.97	10.98	2.804	189.446	1.5841	0.172	-0.150
73	BDE-85	2,2',3,4,4'-pentabromodiphenyl ether	$C_{12}H_5Br_5O$	11.66	11.27	11.28	11.73	11.28	11.50	11.51	1.989	191.508	1.5697	0.173	-0.163
87	BDE-99	2,2',4,4',5-pentabromodiphenyl ether	$C_{12}H_5Br_5O$	11.28	11.18	11.19	11.42	11.19	11.30	11.31	1.411	190.625	1.2624	0.185	-0.161
88	BDE-100	2,2',4,4',5-pentabromodiphenyl ether	$C_{12}H_5Br_5O$	11.13	11.10	11.11	11.15	11.11	11.13	11.13	2.578	189.997	1.3641	0.178	-0.156
107	BDE-119	2,3,4,4',6-pentabromodiphenyl ether	$C_{12}H_5Br_5O$	11.52	10.98	10.99	11.46	10.98	11.22	11.22	1.869	187.696	1.3556	0.178	-0.157
114	BDE-126	3,3',4,4',5-pentabromodiphenyl ether	$C_{12}H_5Br_5O$	11.97	11.29	11.31	11.80	11.30	11.55	11.55	1.922	191.763	1.5558	0.182	-0.157
141	BDE-153	2,2',4,4',5,5'-hexabromodiphenyl ether	$C_{12}H_4Br_6O$	12.15	12.03	12.05	12.06	12.04	12.04	12.05	0.282	205.501	1.3706	0.185	-0.180
142	BDE-154	2,2',4,4',5,6-hexabromodiphenyl ether	$C_{12}H_4Br_6O$	11.92	12.09	12.11	11.94	12.10	12.02	12.03	1.845	204.151	1.4430	0.181	-0.180
144	BDE-156	2,3,3',4,4',5-hexabromodiphenyl ether	$C_{12}H_4Br_6O$	11.98	12.06	12.08	11.96	12.07	12.01	12.02	1.725	206.402	2.0291	0.182	-0.158
172	BDE-184	2,2',3,4,4',6,6'-heptabromodiphenyl ether	$C_{12}H_3Br_7O$	11.96	12.78	12.81	11.96	12.79	12.37	12.38	1.726	219.146	1.8575	0.183	-0.176

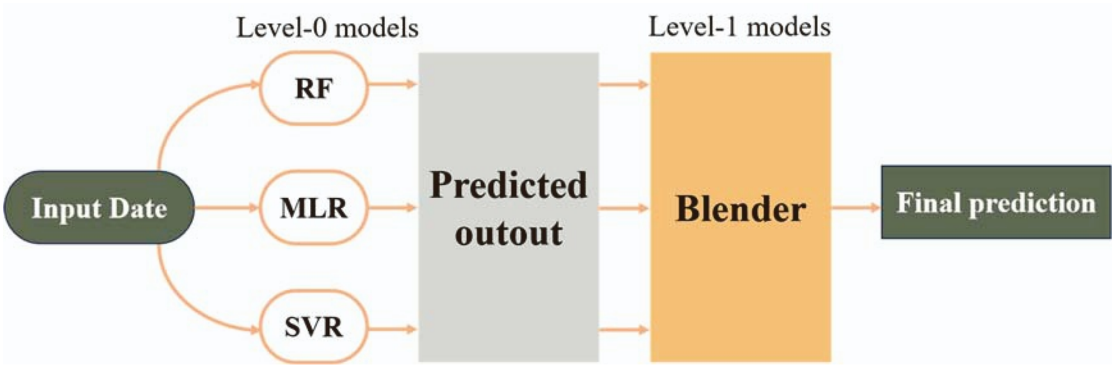

Figure S1. Schematic diagram of the Stacking Ensemble Learning framework employed in this study. (Level-0: Base models trained on original data; Predictions from Level-0 models form new features for Level-1; Level-1: Meta-learner trained on Level-0 predictions to produce final ensemble output).

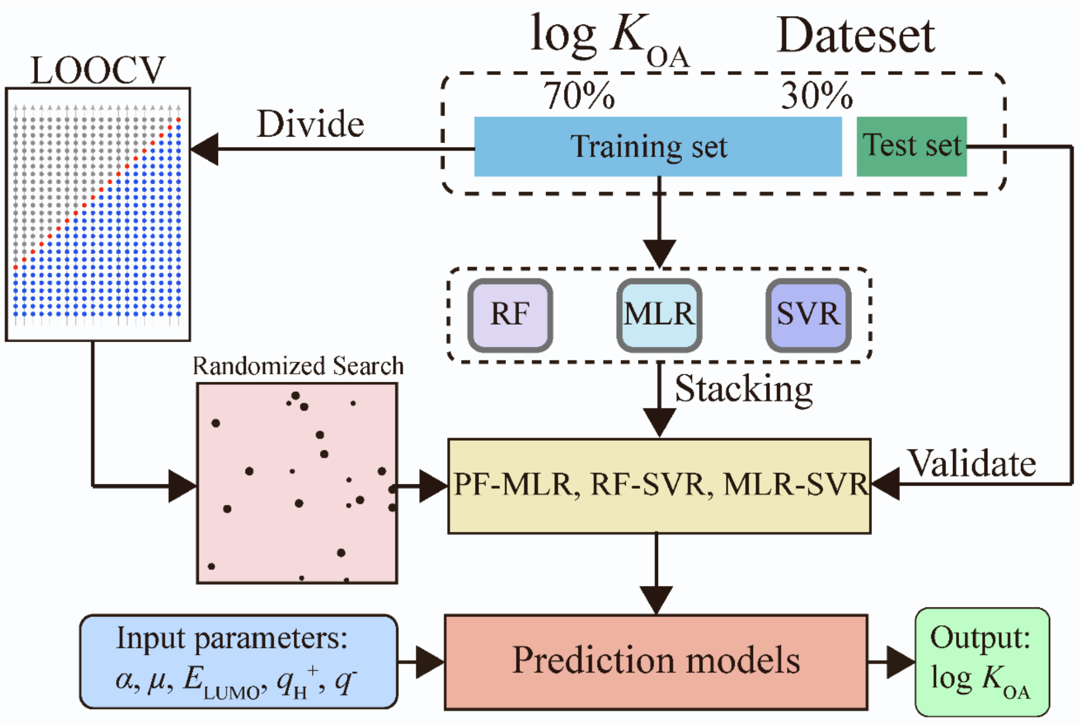

Figure S2. Schematic of the ensemble model training and prediction framework. (Includes data preprocessing, base model training with hyperparameter optimization via LOOCV, Stacking integration, model evaluation, and application).
